# The global pharmacy workforce: a systematic review of the literature

**DOI:** 10.1186/1478-4491-7-48

**Published:** 2009-06-19

**Authors:** Nicola Hawthorne, Claire Anderson

**Affiliations:** 1 Division of Social Research in Medicines and Health, School of Pharmacy, University of Nottingham, Nottingham, UK

## Abstract

The importance of health workforce provision has gained significance and is now considered one of the most pressing issues worldwide, across all health professions. Against this background, the objectives of the work presented here were to systematically explore and identify contemporary issues surrounding expansion of the global pharmacy workforce in order to assist the International Pharmaceutical Federation working group on the workforce.

International peer and non-peer-reviewed literature published between January 1998 and February 2008 was analysed. Articles were collated by performing searches of appropriate databases and reference lists of relevant articles; in addition, key informants were contacted. Information that met specific quality standards and pertained to the pharmacy workforce was extracted to matrices and assigned an evidence grade.

Sixty-nine papers were identified for inclusion (48 peer reviewed and 21 non-peer-reviewed). Evaluation of evidence revealed the global pharmacy workforce to be composed of increasing numbers of females who were working fewer hours; this decreased their overall full-time equivalent contribution to the workforce, compared to male pharmacists. Distribution of pharmacists was uneven with respect to location (urban/rural, less-developed/more-developed countries) and work sector (private/public). Graduates showed a preference for completing pre-registration training near where they studied as an undergraduate; this was of considerable importance to rural areas. Increases in the number of pharmacy student enrolments and pharmacy schools occurred alongside an expansion in the number and roles of pharmacy technicians. Increased international awareness and support existed for the certification, registration and regulation of pharmacy technicians and accreditation of training courses. The most common factors adding to the demand for pharmacists were increased feminization, clinical governance measures, complexity of medication therapy and increased prescriptions.

To maintain and expand the future pharmacy workforce, increases in recruitment and retention will be essential, as will decreases in attrition, where possible. However, scaling up the global pharmacy workforce is a complex, multifactorial responsibility that requires coordinated action. Further research by means of prospective and comparative methods, not only surveys, is needed into feminization; decreasing demand for postgraduate training; graduate trends; job satisfaction and the impact of pharmacy technicians; and how effective existing interventions are at expanding the pharmacy workforce. More coordinated monitoring and modelling of the pharmacy workforce worldwide (particularly in developing countries) is required.

## Introduction

Shortages of pharmacists have been reported in specific countries since the early 1990s. Reports of shortages of the health workforce had surfaced in the 1970s but it was not until the following decade, and in particular the publishing of the *World health report *in 2006 [1], that health workforce issues gained sufficient momentum to merit widespread investigation and international action to bring about changes. That report was a major driving force for expansion of the international health workforce in order to meet the health-related Millennium Development Goals. The Global Health Workforce Alliance was established to accelerate progress towards these goals by identifying and implementing solutions to the shortages [2]. The international shortage of health care professionals exists in different severities and has different root causes, depending on the particular health profession and the country of origin. Health care priorities therefore change between countries: a universal health system would invariably not provide the required health care efficiently to all those who need it. The *Global pharmacy workforce and migration report *was the first of its kind to investigate specific workforce issues affecting the international pharmacy profession as a whole [3].

This review focuses upon the issues facing the expansion of the global pharmacy workforce; by gathering together past and present literature, it provides a platform for discussion, planning and action to enable the management of current problems and the foresight of future challenges worldwide. The main objectives of this report are to systematically identify and review the contemporary issues surrounding the global pharmacy workforce and, more specifically, to explore the published methods used to expand the workforce. The review was produced for the International Pharmaceutical Federation's working group on the pharmacy workforce.

## Methods

Relevant peer-reviewed and non-peer-reviewed international literature was initially identified via searches on electronic databases. The databases searched included MEDLINE, EMBASE, International Pharmaceutical Abstracts, PubMed and The Cochrane Library. The search terms used were "pharmacy workforce", "pharmacy manpower", "human resources for health AND pharmacy", "human resources AND pharmacy" and "pharmacist shortage". Members of the International Pharmaceutical Federation working group on the pharmacy workforce provided country-specific literature on Canada and the United States of America. In addition, reference lists of relevant articles were searched. Copies of all the evidence included in the review were obtained.

The criteria for inclusion were that the literature related to pharmacists, pharmacy technicians or pharmacy assistants from any country worldwide; was published between January 1998 and February 2008; and that it satisfied the Health Development Agency Evidence Base 2000 standards [4] (with some noted exceptions). The review excluded workforce imbalances within pharmacy specialties (such as the mental health pharmacy workforce); literature published relating to historical data; non-English language literature; human resource matters concerned with delivering therapy for specific diseases (for example HIV and AIDS); and workforce issues surrounding emergency situations (such as natural disasters, conflict and epidemics). Once the relevant papers and reports were identified for inclusion, each document was ascribed an evidence grade used by the Department of Health in National Service Frameworks [5] and key data relating to the pharmacy workforce were extracted to matrices (see Additional files 1 and 2, which were independently checked by the second author). The evidence that did not meet all the Evidence Base 2000 standards was clearly annotated in the matrices.

## Results

In total, 69 papers were identified for inclusion in the review: 48 peer-reviewed papers and 21 non-peer-reviewed reports. Most of the evidence gathered was in the B3 category (individual, well-designed, non-experimental studies; well-designed qualitative studies; and well-designed analytical studies, including secondary analysis), which was also the highest evidence grade achieved in this review. The papers and reports revealed several key areas important in workforce planning and expansion; these are detailed below.

### Demographics

The proportion of females within the pharmacy workforce was found to either predominate, as observed in the United Kingdom [6], Canada [7], New Zealand [8] and Ireland [9], or be increasing, as seen in the United States between 2000 and 2004 [10,11]. The age of practising pharmacists was another important demographic issue presented in the national pharmacist workforce data from these countries. In general, the largest proportion of pharmacists was aged between 30 and 45 years [6,11-16] and the majority of male pharmacists tended to be older than the females; this was the case in New Zealand [8], the United Kingdom [6], the United States [11], Ireland [9], Australia [14] and Canada [7]. Generally male pharmacists predominated above the age of 50.

### Education

One response to the shortage of pharmacists was found to be a planned expansion of the number of pharmacy graduates, which occurred or was recommended in the United Kingdom [17], the United States [14], Australia [12], Canada [15], Ireland [9] and Northern Ireland [16]. Expansion was indicated by an increase in the number of pharmacy schools or increases in enrolments at existing schools or increased numbers of entrants to the profession. However, this expansion presented many concerns regarding quality of teaching, the number of available pharmacy-trained faculty and the academic standard of applicants. In addition, alignment of pharmacy curricula with pharmacy practice was considered important for job satisfaction [18-22] and hence retention of pharmacists.

### Distribution

Four important types of distribution became apparent within the pharmacy workforce: urban and rural; private sector and public sector; international migration; and movement between workplace sectors. Distribution of pharmacists was found to be uneven, with fewer pharmacists employed relative to population in rural or remote locations, compared with urban environments [8,22,23]; public or federal sector posts were less likely to be filled, compared with private sector positions [24-26]; and there was greater migration from less-developed countries to more-developed countries The pharmacist workforce of African countries was disproportionately affected by these trends [22]. Graduates also showed a preference for completing pre-registration training near where they studied as an undergraduate [27,28]; this was of considerable importance when planning recruitment to rural areas in Australia [23].

### Pharmacy technicians

The relative importance of pharmacy technicians within the contemporary pharmacy workforce has been amplified, largely as a reaction to pharmacist shortages. As such, their numbers and responsibilities have been increased [29-31]. There was also found to be increased international awareness and support for the certification, registration and regulation of pharmacy technicians, and accreditation of the relevant training courses [32-34].

### Feminization

The aforementioned increased proportion of female pharmacists in many countries brought to light specific issues surrounding their work patterns, particularly workforce participation. The prevalence of part-time work among female pharmacists was found to be much greater than that of their male counterparts in several countries [35,36], and as a result the full-time equivalent contribution of females was lower than that of males [36]. Females were found to be overrepresented in the hospital sector [11,9,20,35] and underrepresented in higher-status roles such as management in the United States [11] and the United Kingdom [35]. The number of female pharmacy students graduating was also noted to have increased, thus giving weight to the fact that female workforce issues will become increasingly important in the future. Reports of females comprising approximately two thirds of all pharmacy graduates were not uncommon [7,9,25,37,38].

### Graduate trends

Graduate trends were important to investigate, as they may be used to predict and prepare for future workforce planning issues. A large proportion of pharmacy graduates in the United Kingdom intended to take a career break [39], and as mentioned earlier, graduates also showed a preference to complete pre-registration training near where they studied as an undergraduate. The university at which undergraduate training was completed in the United Kingdom was also revealed to potentially influence in which sector of pharmacy graduates decided to pursue their future careers [40]. Growing numbers of young pharmacists and pharmacy graduates originated from ethnic minorities in the United Kingdom [41].

### Job satisfaction

Job satisfaction was viewed as an important indicator of staff turnover and retention. Factors identified as increasing pharmacist retention in the United States were good remuneration, good relationships with co-workers and flexible schedules. Factors increasing staff turnover included high stress, insufficient or unqualified staff and poor salary [42].

### Supply and demand factors

Increased demand or limited supply of pharmacists constrains the ability of the workforce to expand. Many different supply and demand factors that influenced the pharmacy profession were identified, the majority of which were common to most countries. The most common factors increasing demand for pharmacists were increased feminization, increased clinical governance measures through continually reviewing and improving the quality of patient care, increased numbers of prescriptions and increased complexity of medication therapy. The most common factors mitigating demand for pharmacists included increased use of technology, expansion in the numbers and roles of pharmacy technicians and increased numbers of pharmacy graduates [9,20,15,21,39-41].

## Discussion

Most of the papers identified for inclusion were judged to be of sound methodological quality and each added value to understanding the factors surrounding the expansion of the pharmacy workforce. The issues surrounding planning and expansion of the pharmacy workforce elucidated from the literature will be discussed in relation to recruitment, retention and attrition.

### Recruitment

**Figure 1:**
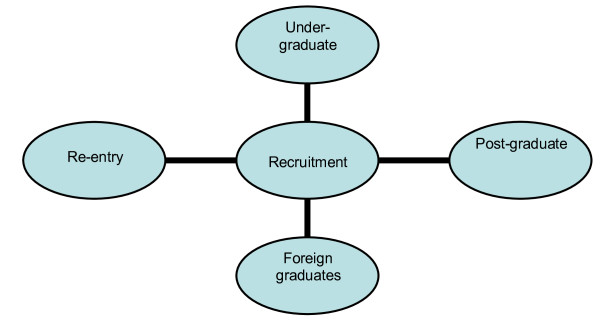
**Potential areas of recruitment to the pharmacist workforce**.

There are four relatively distinct areas of recruitment, as seen in Figure 1, which may be relied upon as routes to expand the pharmacy workforce: undergraduate, postgraduate, re-entry and foreign pharmacy graduates. The first of these, undergraduate recruitment, is the only process that will lead to expansion of the overall number of qualified pharmacists. The literature suggested that the main methods used to increase the number of qualified pharmacists was to expand the number of students enrolled in current pharmacy courses and increase the overall number of pharmacy courses.

Maintaining the quality and prestige of the pharmacy profession by retaining high-quality applicants was viewed with great importance; measures should be undertaken to increase the applicant pool in order to select the best candidates for pharmacy. Nevertheless, it seems inevitable that if enrolments increase significantly, a lower academic standard of pharmacists will result, since if student intake keeps increasing but the pool of potential students does not, schools may have to take applicants with lower entrance qualifications. The academic standards at which the course is set will probably not be achieved by less capable individuals (unless these standards are lowered), increasing the possible numbers who drop out of the course or those unable to pass a licensing exam where one exists.

Also, a United Kingdom report noted that pharmacy enrolments may be adversely affected by the increase in the number of medical school positions, with the medical profession similarly trying to increase enrolments to redress shortages [17]. Therefore, expansions in the number of alternative science-based degree courses may also be a factor limiting the expansion of suitable applicants to pharmacy.

Another important issue in the recruitment of pharmacists was the lack of male students entering the pharmacy degree course; the workforce implications of having a high female component have been extensively relayed. However, the reasons why males and females choose to study pharmacy or choose not to study pharmacy remain unknown.

There was a lack of pharmacy students choosing to undertake postgraduate pharmacy education. As academics are usually required to hold a postgraduate degree, this may worsen the pharmacy faculty shortages identified in both the United Kingdom and United States literature. A decline in the pharmacist-to-student ratio or a reduced rate of expansion may result if more pharmacist faculty were not recruited.

Another valuable area for pharmacy recruitment is the current inactive or part-time workforce. However, the literature indicates that the capacity for increasing the participation of this proportion of the workforce is minimal, either because of the high proportion of female pharmacists with family responsibilities, the high desirability of career breaks and part-time hours or the increasingly early age of (phased) retirement.

The final route of increasing the size of one particular country's pharmacy workforce is to recruit from another country's pool of pharmacy graduates, which can be inherently controversial. The increasing migratory flow of the health care workforce was of particular concern in developing countries, as the majority of migrating pharmacists moved to more-developed countries. While this was seen to benefit the individual for a variety of reasons, when emigration occurred disproportionately it severely hampered the provision of adequate health care to the home nation. Nevertheless, despite the human resource crisis in developing countries the opinion acknowledged by this investigation was not to prevent the flow of migration (partly due to the importance of remittances received by the families of expatriates) but instead to emphasize the need for exchange of professional expertise.

### Retention

**Figure 2:**
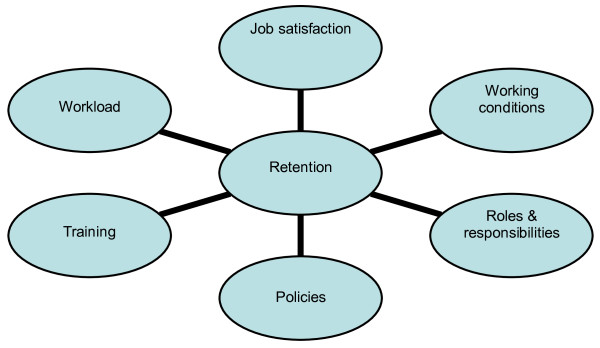
**Factors affecting pharmacist retention**.

Retention was frequently reported as being a problem and a number of reasons, illustrated in Figure 2, were identified as being partly responsible for these difficulties. The first to be discussed is the effect of job satisfaction on retention. A theme echoed throughout the literature studied was that alignment of career expectations, aptitude and the pharmacy course content with the actual realities of practising pharmacy was imperative to ensure career satisfaction. Another key issue revealed by the literature regarding pharmacy curricula was that the curricula taught in developing countries were similar to those of developed countries. While this produced highly competent individuals, it did not necessarily prepare them for the realities of a career in their own country, thus disillusionment and frustration may result in increased emigration to more-developed countries, facilitated by the similarity of the degree course. In order to model the demands for pharmacists, it is very important to define needs-based roles for all cadres in the pharmacy workforce in any particular country. In addition, evolving, new and emerging technologies and innovative practice models and their impact on the workforce must be described for particular country and health systems.

Training and career advancement were also predominant in the literature, especially surrounding the retention of female pharmacists and pharmacy technicians. Female pharmacists, although making up the majority of the workforce, were underrepresented in management positions, which was shown to be a result of their personal choices influenced by family responsibilities in the United Kingdom [42]. In terms of the pharmacy technician workforce, the lack of a "career ladder" or opportunities for career progression was the most frequent cause of dissatisfaction.

While wider roles were generally welcomed by pharmacists as a chance to make use of a greater breadth of their training, it may also be prudent to mention the potential of role overload, which may result due to high expectations for service delivery, unless sufficient resources and staffing occur simultaneously or a shifting of roles and responsibilities occurs.

Working conditions and workload were also shown to have a significant impact on retention, encompassing a wide range of intrinsic and extrinsic factors. Only a limited number of factors adversely affecting working conditions and workload can be tackled by individual employers, but wider-ranging alterations may call for changes in government legislation or company policy.

### Attrition

**Figure 3:**
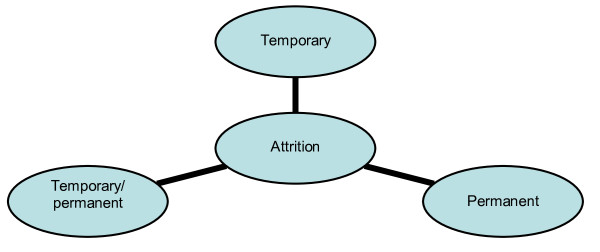
**Forms of attrition in the pharmacy workforce**.

The loss of participating pharmacists from the workforce needs to be taken into account to obtain a more accurate understanding about the net change in size of the workforce. As seen in Figure 3, three broad forms of attrition were identified from the literature as temporary, temporary or permanent, and permanent loss.

In the case of temporary removal from active participation in the workforce, the most significant factor seemed to be the high preference for career breaks within the pharmacy profession. The reasons for this should be explored further. However, it may be postulated that with increasing proportions of female pharmacists present in the workforce, more females will take time off to raise a family. This may even be facilitated by the growing numbers of chain pharmacies, as they are likely to have greater capacity to support maternity or paternity leave, compared to independent owner/manager pharmacies.

Another factor involved in temporary attrition of the workforce is involvement in training courses. When pharmacists, pharmacy technicians or pharmacy assistants are engaged in a training course, they are not providing a service; unless these courses take place outside the hours of normal work, they reduce the capacity of the workforce to expand, as substitutes will be required to fill the temporarily vacant positions. This factor is likely to grow in significance with the sustained emphasis on continuing professional development, continuing education and risk management measures.

Factors affecting the loss of participation in the pharmacy workforce that may be either temporary or permanent were classified as part-time working and migration. The increasing trend of part-time working was largely due to the increased proportion of female pharmacists, but it was noted in the United States that the number of male pharmacists working part-time also increased between 2000 and 2004. This may not be part of a growing trend, but nonetheless this situation should be monitored.

A possible reason for increased part-time working among males may be increased salaries due to pharmacist shortages, making part-time working more economically viable. But perhaps the most likely reason may have been the self-implementation of phased retirement, as the majority of male pharmacists were in the older age groups. Nevertheless, increased part-time working, whether undertaken by male or female pharmacists, is a concern for workforce expansion, as more pharmacists will be needed to maintain current levels of service provision due to reduced pharmacist full-time-equivalent contributions.

International migration of pharmacists can result in a net loss or gain of pharmacists. The exchange of knowledge and skills is valuable, but large or continuous net losses can have serious detrimental effects on the source workforce. In order to minimize the potential damage while maximizing the advantages, a sound understanding of pharmacist migration must be achieved.

Migration is being accelerated by workforce shortages. When there are shortages, pharmacists are pushed back into the dispensaries and away from direct patient care – for which they are prepared by undergraduate courses – towards largely supply roles. The relevance of continuing education courses is then questionable because of the lack of capacity to integrate new knowledge and skills into the workplace. These circumstances accelerate the move from the public sector to the private sector and to emigration.

Permanent loss from the workforce – true attrition – was attributed to changing employment to a field outside pharmacy, retirement or death. Not much was known about the numbers of qualified pharmacists working outside pharmacy, as unless they remain registered there is no way of tracking them. However, a factor increasing the demand for pharmacists was the movement of pharmacists into non-traditional areas of work. The identification of this trend clearly meant that pharmacists involved in these fields remained registered. Nevertheless, if this set of circumstances changes, leakage of pharmacists to "other" employment sectors may go unnoticed.

Retirement can also only be estimated, as retired, inactive pharmacists do not legally have to remain registered. However, those who do and are over the state pension age have provided very interesting information about the pharmacy workforce. A development of concern was that male pharmacists were generally predominant in the workforce by a considerable margin after the age of 50; considering that male pharmacists in the overall workforce were in the minority, it appears that female pharmacists leave the profession much younger than their male counterparts. Despite this, the majority of pharmacists were found to be aged between 30 and 45 years. Therefore, as long as adequate numbers of newly qualified pharmacists and pharmacy technicians enter the workforce to maintain the high proportion of the workforce in younger age groups, pharmacy should not be expected to become an ageing profession.

Finally, the death of pharmacists was another factor in the permanent attrition of pharmacists from the workforce. Although the death of pharmacists was not reported to be a problem in any of the literature included in the review, most of the literature was from developed countries with relatively low death rates compared to less-developed countries. However, the *World health report *in 2006 revealed that deaths due to HIV/AIDS were alarmingly numerous within the health workforce in several African countries [1]. This raises the question: If health care professionals cannot get access to effective treatment, what hope does the rest of the population have?

### Limitations

This review of literature found a significant amount of information detailing the characteristics of the pharmacy workforce in developed countries. However, there were significant shortfalls of published information regarding the pharmacist workforce in developing nations and also that relating to the effectiveness of any interventions used to expand the pharmacy workforce. Although this does limit the generalizability of this review, it does not devalue its usefulness. It also provides several comparators for additional research in the excluded countries. There was also a shortfall of literature relating specifically to the global pharmacist workforce as a whole: the only other international report on the pharmacy workforce was the Global Pharmacy Workforce Report [3] commissioned by the International Pharmaceutical Federation, the second edition of which is currently being produced.

### Research implications

Most of the evidence included in this review is derived from surveys and is rated at a relatively low level. Future prospective and comparative research might use observational methodologies for certain aspects such as graduate trends, job satisfaction and the impact of pharmacy technicians. Further research into why males are increasingly choosing not to study pharmacy and a more coordinated monitoring of the pharmacy workforce worldwide (particularly in developing countries) are needed. Also, research into why pharmacy students are increasingly not pursuing postgraduate education and what measures can be taken to encourage careers in academia should be undertaken.

## Conclusion

This review adds significantly to the current understanding of the international pharmacy workforce by bringing together and evaluating the relevant literature from around the world. To maintain and expand the future pharmacy workforce, increases in recruitment and retention will be essential, as will decreases in attrition where possible. However, scaling up the global pharmacy workforce is a complex, multifactorial responsibility that requires coordinated action. The repercussions of any changes made to the pharmacy workforce need to be considered carefully and optimal use of the current workforce should be made.

## Competing interests

The authors declare that they have no competing interests.

## Authors' contributions

NH carried out this study as part of her MPharm degree. CA independently reviewed all the papers and commented on each draft of the paper.

## Supplementary Material

Additional file 1 **Table 1. Record of peer-reviewed evidence**. Annotated references. Click here for file

Additional file 2 **Table 2. Record of non-peer reviewed evidence**. Annotated references. Click here for file
