# Supplementary material for: The global pharmacy workforce: a systematic review of the literature
Source: Hum Resour Health. 2009 Jun 19;7:48. doi: 10.1186/1478-4491-7-48 (PMC2706790; doi:10.1186/1478-4491-7-48)
Supplement: Additional file 1 — Table 1. Record of peer-reviewed evidence. Annotated references. [file 1478-4491-7-48-S1.doc]

### Table 1. Record of peer-reviewed evidence

* Denotes that funding was not explicitly declared. † Denotes that no methodology was described.

| **Reference** | **Methodology, research design & evidence category** | **Key findings** |
| --- | --- | --- |
| **Pharmacist participation in the workforce: 1990, 2000, and 2004**  Mott DA, Doucette WR, Gaither CA, Kreling DH, Pedersen CA, Schommer JC  *Journal of the American Pharmacists Association* 2006, **46**(3):322-330  USA | Three cross-sectional, descriptive studies based on data collected from national studies of the pharmacist workforce conducted in 1990, 2000 and 2004.  B3 | Over 86% of pharmacists were actively practising pharmacy at the time of these surveys.  There has been a reduction in pharmacists’ participation in the workforce between 2000 and 2004, as illustrated by a decrease in the full-time equivalent (FTE) contribution, which decreased between 2000 and 2004: 0.87 to 0.81 for women and 0.99 to 0.91 for men.  The proportion of pharmacists working part-time has increased in each year of the survey, from 16% in 1990 to 20.6% in 2004.  The 31–45 age group contained the highest proportion of male pharmacists in 1990, but in 2004 this was true of the 46–60 age group. |
| **Community pharmacists’ work environments: evidence from the 2004 national pharmacist workforce study**  Kreling DH, Doucette WR, Mott DA, Gaither CA, Pedersen CA, Schommer JC  *Journal of the American Pharmacists Association* 2006, **46**(3):331-339  USA | Cross-sectional study of community pharmacists (independent, chain, mass merchandiser and supermarket settings) based on data collected from the 2004 National Pharmacist Workforce Survey and compared with data obtained from the 2000 National Pharmacist Workforce Survey where possible.  B3 | Pharmacists were working with more pharmacy technicians in 2004 compared to 2000.  The number of prescriptions personally dispensed per pharmacist per day increased significantly between 2000 and 2004 across all settings.  The effect of workload on motivation and job satisfaction received the most positive ratings, whereas the opportunity to take adequate breaks received the majority of negative ratings.  Equipment used in dispensing was more common than that used in patient care. Interestingly, approximately half of the pharmacists reported a “neutral” effect of equipment and technology on time spent on dispensing. |
| **Pharmacists’ desired and actual times in work activities: evidence of gaps from the 2004 National Workforce Study**  Schommer JC, Pedersen CA, Gaither CA, Doucette WR, Kreling DH, Mott DA  *Journal of the American Pharmacists Association* 2006, **46**(3):340-347  USA | Cross-sectional study of pharmacies in the USA, using data collected from the 2004 National Pharmacist Workforce Survey, which was compared with data extracted from the 2000 National Pharmacist Workforce Survey where applicable.  B3 | Pharmacists in chain and independent settings were more likely to report discordance between desired and actual time spent in certain work activities compared with those in hospital or other patient care.  Working with other pharmacists or having a higher proportion of pharmacy staff did not appear to alter the desired or actual time spent on the measured work activities.  Practice settings were found to be the most influential factor concerning pharmacists’ work activities.  Across all practice settings there was a desire to spend more time on consultation and drug use management and less time on medication dispensing. |
| **Evaluation of community pharmacy service mix: evidence from the 2004 National Pharmacist Workforce Study**  Doucette WR, Kreling DH, Schommer JC, Gaither CA, Mott DA, Pedersen CA  *Journal of the American Pharmacists Association* 2006, **46**(3):348-355  USA | Cross-sectional study of community pharmacy settings (independent, chain, mass merchandiser and supermarket) using the data obtained from the 2004 National Workforce Survey.  B3 | The most common product-related service provided was general/simple compounding. Four pharmacy care services; diabetes management, immunization, smoking cessation and health screening were offered in more than 10% of pharmacies. The most widespread information service available was a drug information service.  54.4% of the community pharmacies did not offer any pharmacy care services. Only 10.1% of pharmacies reported receiving payment for non-dispensing services. A positive correlation was discovered between the number of pharmaceutical care services available in community pharmacies; when there were at least three pharmacists on duty, the innovativeness of the pharmacy and the status as an independent or supermarket pharmacy. |
| **Should I stay or should I go? The influence of individual and organisational factors on pharmacists’ future work plans**  Gaither CA, Nadkarni A, Mott DA, Schommer JC, Doucette WR, Kreling DH, Pedersen CA  *Journal of the American Pharmacists Association* 2007, **47**(2):165-173  USA | Cross-sectional study using data collected from the 2004 National Pharmacist Workforce Survey.  B3 | The future work plans of 15% of respondents were to leave their current employer within the next year.  More than half of the respondents perceived their workload as high to excessively high and that it had increased over the past 12 months.  Logistic regression analysis showed that unmarried individuals were 1.7 times more likely to leave than married respondents, and non-white pharmacists were twice as likely to leave as were white respondents.  When one main factor influenced leaving, commonly it was found to be insufficient and/or unqualified staff; similarly, when one main factor influenced staying, flexible scheduling was the most influential. |
| **A four-state summary of the pharmacy workforce**  Mott DA, Sorofman BA, Kreling DH, Schommer JC, Pedersen CA  *Journal of the American Pharmaceutical Association* 2001, **41**:693-702  USA | A cross-sectional descriptive survey design was used to collect data on pharmacists in Ohio, Iowa, Minnesota and Wisconsin. Survey participants were selected by means of random selection in Ohio and systematic random selection in Minnesota, Iowa and Wisconsin. The number of pharmacists surveyed was relative to the resources available in each state. The survey was 12 pages in length, except surveys in Iowa, which were 4 pages, but there were identical core questions in the surveys administered in the other three states.  B3 | A response rate of 52.4% was achieved.  The majority of respondents were male (56%).  24.2% of females worked less than 31 hours per week, compared with 6.3% of males. Hourly wage rates for part-time and full-time pharmacists were similar.  Management positions accounted for 49% of male pharmacists working full-time, compared to 30.4% of female pharmacists.  The largest proportion of women was aged between 31 and 45 years, whereas the largest proportion of men was aged 46 to 60 years.  Out of actively participating pharmacists, 11.4% held an additional job as a pharmacist. |
| **ACCP white paper: a vision of pharmacy’s future roles, responsibilities and manpower needs in the United States**†  Maddux MS, Dong BJ, Miller WA, Nelson KM, Raebel MA, Raehl CL, Smith WE  *Pharmacotherapy* 2000, **20**(8):991-1020  USA | A report compiled over two years containing observations, analyses and recommendations regarding the future roles and workforce requirements of the USA pharmacy workforce.  D | Key findings relating to future pharmacy manpower:  A shortage or a surplus may result in the USA future pharmacy workforce; dependent upon the extent of increased efficiency in drug distribution with minimum pharmacist engagement and adaptation of the profession to engage in patient care activities.  Various factors were identified as either amplifying or diminishing demand for pharmacists.  There was a shortage of pharmacy faculty highlighted due to the increased number of pharmacy schools and students, the static number of faculty training programmes and an increased number of pharmacy faculty taking up employment in industry.  Increased efforts to recruit more individuals into academic careers were advocated. |
| **Pharmaceutical education and the pharmacy workforce. Should we expand our programs? Report of the AACP Argus Commission 1999-2000** *†  Cohen JL, Kabat HF, Knapp DA, Koda-Kimble MA, Rutledge CO  *American Journal of Pharmaceutical Education* 2000, **64**:4S-7S  USA | A report by the Argus Commission outlining pharmacy workforce issues and how these affect pharmacy education. The Argus Commission consisted of the five most recent past presidents of the American Association of Colleges of Pharmacy (AACP).  D | Scaling up pharmacy education by either commissioning new schools or expanding existing schools of pharmacy was recommended to meet an increased demand for pharmacy services.  The need and ability for the expansion of pharmacy education was recognized to be determined by local factors.  The number of pharmacy applicants has declined since 1995.  The shortage of academic pharmacists necessitates enhanced promotion of careers in this area.  There was a perceived need for more emphasis on the responsibilities of managing dispensing and drug distribution, which was seen to be overshadowed by clinical training. |
| **ASHP national survey of pharmacy practice in hospital settings: Prescribing and transcribing – 2001**  Pederson CA, Schneider PJ, Santell JP  *American Journal of Health-System Pharmacy* 2001, **58**(23):2251-2266  USA | A questionnaire was designed and administered to pharmacy directors at 1091 general and children’s medical-surgical hospitals. This sample of 1091 hospitals was randomized and stratified by hospital size.  B3 | A response rate of 49.0% was achieved.  Key findings relating to staffing:  Averages of 9 FTE pharmacists and 8 FTE pharmacy technicians per hospital were established, but these varied with respect to hospital size.  The ratio of pharmacists to technicians was similar across all hospital sizes (range, 1.02 to 1.19). This ratio was described as suboptimal. |
| **ASHP national survey of pharmacy practice in hospital settings: Dispensing and administration – 2002**  Pederson CA, Schneider PJ, Scheckelhoff DJ  *American Journal of Health-System Pharmacy* 2003, **60**(1):52-68  USA | A questionnaire was designed and administered to pharmacy directors at 1101 general and children’s medical-surgical hospitals. This sample of 1101 hospitals was randomized and stratified by hospital size.  B3 | A response rate of 46.7% was achieved.  Key findings relating to staffing:  The average number of FTE pharmacy technicians per hospital was 8, but this varied depending on hospital size.  There was an 8.5% decrease in pharmacy staffing between 2001 and 2002. An estimated vacancy rate of 7% was established.  There was a decline in pharmacist-to-technician ratio, suggesting greater use of pharmacy technicians. |
| **ASHP national survey of pharmacy practice in hospital settings: Monitoring and patient education – 2003**  Pederson CA, Schneider PJ, Scheckelhoff DJ  *American Journal of Health-System Pharmacy* 2004, **61**(5):457-471  USA | A questionnaire was designed and administered to pharmacy directors at 1173 general and children’s medical-surgical hospitals. This sample of 1173 hospitals was randomized and stratified by hospital size.  B3 | A response rate of 47.1% was achieved.  Key findings relating to staffing:  The number of FTE pharmacists per hospital rose substantially from the previous year, from an average of 8.6 FTEs in 2002 to 9.4 FTEs in 2003. The average number of FTE pharmacy technicians was 8.6 per hospital, but this varied with hospital size.  Pharmacist vacancy rate decreased from 7.3% in 2002 to 4.3%, which correlated to an estimated 1846 FTE pharmacist vacancies in hospitals in 2004. |
| **ASHP national survey of pharmacy practice in hospital settings: Prescribing and transcribing – 2004**  Pederson CA, Schneider PJ, Scheckelhoff DJ  American Journal of Health-System Pharmacy 2005, **62**(4):378-390  USA | A questionnaire was designed and administered to pharmacy directors at 1183 general and children’s medical-surgical hospitals. This sample of 1183 hospitals was randomized and stratified by hospital size. Non-responders were contacted up to a maximum of six times.  B3 | A response rate of 41.7% was achieved.  Key findings relating to staffing:  The number of FTE pharmacists and FTE pharmacy technicians per hospital varied significantly by hospital size but averaged 9.8 and 9.1, respectively.  The FTE pharmacy vacancy level was reported to be 6.3%, which correlated to an estimated 3085 FTE pharmacist vacancies in hospitals in 2004. |
| **ASHP national survey of pharmacy practice in hospital settings: Dispensing and administration – 2005**  Pederson CA, Schneider PJ, Scheckelhoff DJ  *American Journal of Health-System Pharmacy* 2006, **63**(4):327-345  USA | A questionnaire was designed and administered to pharmacy directors at 1173 general and children’s medical-surgical hospitals. This sample of 1173 hospitals was randomized and stratified by hospital size. Non-responders were contacted up to a maximum of six times.  B3 | A response rate of 43.5% was achieved.  Key findings relating to staffing:  The number of FTE pharmacists and FTE pharmacy technicians per hospital varied significantly by hospital size but averaged 10.1 and 9.7, respectively. These staffing levels correlated with increasing numbers of pharmacists and pharmacy technicians in the previous three years.  The proportion of vacant FTE pharmacist positions was estimated to be 5.6%, which correlated to an estimated 2759 vacancies at any one time in 2005. |
| **ASHP national survey of pharmacy practice in hospital settings: Monitoring and patient education – 2006**  Pederson CA, Schneider PJ, Scheckelhoff DJ  *American Journal of Health-System Pharmacy* 2007, **64**(5):507-520  USA | A questionnaire was designed and administered to pharmacy directors at 1178 general and children’s medical-surgical hospitals. This sample of 1178 hospitals was randomized and stratified by hospital size. Non-responders were contacted up to a maximum of six times.  B3 | A response rate of 39.0% was achieved.  Key findings relating to staffing:  The number of FTE pharmacists and FTE pharmacy technicians per hospital varied significantly by hospital size but averaged 9.8 and 9.0, respectively.  An average of 4.6% of FTE pharmacist positions were vacant. |
| **Challenges to the pharmacist profession from escalating pharmaceutical demand**†  Cooksey JA, Knapp KK, Walton SM, Cultice JM  *Health Affairs* 2002, **21**(5):182-188  USA | Journal article that presented data describing the pharmacist shortage, responses to the shortage and pharmacists’ expanding roles.  C1 | Increased drug use and spending alongside an increased variety of pharmaceutical employment contributed to increased demand for pharmacists.  Responses to the shortage of pharmacists included increasing the number of pharmacy graduates, thus moderating supply issues, and increasing the use of pharmacy technicians and technology to increase productivity. Increased wages may have attracted new student applicants and the re-entry of pharmacists previously not practising pharmacy.  The expanded roles and responsibilities of pharmacists progressed slowly, especially in communities where a lack of training was a perceived barrier. |
| **Pharmaceutical services in rural hospitals in Illinois – 2001**  Schumock G, Walton S, Sarawate C, Crawford SY  *American Journal of Health-System Pharmacy*  2003, **60**(7):666-674  USA | A questionnaire was designed and mailed to all 71 rural hospital pharmacy directors in Illinois whose acute care facility met the inclusion criteria. Non-responders were contacted on a maximum of five additional occasions.  Non-response bias was analysed.  B3 | Completed questionnaires were returned by 47 participants (response rate of 66%).  A greatly increased proportion of hospitals offered more patient care services in 2001 compared to 1991.  A vacancy rate of 14% for pharmacist positions and 3% for support staff positions was determined.  The most common reasons stated as the cause of vacancies was geographical location and inadequate salary.  In response to vacancies, a reduction in clinical pharmacy services and an expansion in technician roles commonly occurred. Vacancies were also reported to affect job satisfaction of pharmacy staff, reduce physician and nursing satisfaction with pharmacy services and increase pharmacy-related medication errors, among other factors.  Rural hospitals were fewer in number, size and patient volume in 2001 compared with 10 years previously, but no similar decrease in pharmacy staff had occurred. The ratio of pharmacist FTEs to support staff FTEs was 1.0 to 1.08 and the ratio of pharmacist FTEs to operational beds was 1.0 to 22.6. |
| **Assessment of pharmacy manpower and services in West Virginia***  Robinson ET, Bowyer D  *Research in Social and Administrative Pharmacy* 2006, **2**:359-369  USA | A 32-question survey was mailed to a random sample of 548 active pharmacists living in West Virginia; this represented approximately one third of the active register. The survey was followed up with a reminder postcard two weeks after the initial mailing.  B3 | A response rate of 32.8% was obtained.  The gender of respondents was evenly distributed and the proportion working full-time was 78.5%.  Out of the disease management programmes surveyed, diabetes management was most commonly offered (38.4%). It was reported that more pharmacists would like to offer disease management services than the proportion of pharmacists who already provided these services.  Inadequate staffing levels were viewed by 70% of respondents to be the main barrier to implementing disease management services.  The proportions of respondents who reported trying to fill vacant pharmacist and pharmacy technician positions in the last six months was 55.4% and 63.8%, respectively.  There was a perceived shortage of pharmacists and pharmacy technicians in West Virginia by 81.5% and 54% of respondents, respectively. The majority believed the shortage of pharmacists was worse in rural areas. |
| **Update on he national pharmacist shortage: National and state data through 2003***  Knapp KK, Quist RM, Surrey MW, Miller LM  *America Journal of Health-System Pharmacy* 2005, **62**:492-499  USA | 42 panellists representing organizations employing community pharmacists and institutional pharmacists, and integrated health systems with multiple pharmacist positions provide monthly reports on the difficulties of filling pharmacist vacancies in each state. The Aggregate Demand Index (ADI) is then calculated. Time series analysis was conducted on the data provided to identify trends in ADI from September 1999 to September 2003. National and state trends were identified and time trends for the distribution of ratings and the demand index by practice site were also examined.  B3 | A very slight downward trend was observed in the severity of the pharmacist shortage from 1999 to 2003; there were slight decreases in ADI ratings equivalent to excess demand, slight increases in those reporting a balance between supply and demand and a slight increase in ratings reporting excess supply. However, 72% of respondents indicated experiencing excess demand for pharmacists, which was supported by the fact that the majority of the population (88%–98%) lived in areas where filling vacancies was at least moderately difficult.  The least difficulty was experienced in filling community pharmacist vacancies; the greatest difficulty in filling vacancies was reported in organizations.  States with the largest populations generally reported the most severe shortages, and states with smaller populations tended to report lower levels of shortages.  The ADI in 17 states improved, whereas in 30 states it remained the same and in 4 states the ADI worsened between 1999 and 2003. |
| **Predicting the impact of Medicare Part D implementation on the pharmacy workforce**  Meissner B, Harrison D, Carter J, Borrego M  *Research in Social and Administrative Pharmacy* 2006, **2**:315-328  USA | Univariate and multivariate forecasting models were constructed with retrospective data.  The Aggregate Demand Index was the dependent variable. Estimates were calculated for the ADI from January 2006 through December 2009.  Historical data on independent variables, such as prescription volume among others used in the multivariate forecast, were taken from March 2001 through December 2005.  B3 | The ADI had been decreasing slowly towards a balance of supply and demand.  The univariate model predicted that the ADI would continue to decrease, levelling out between 3 and 3.5 by 2009 (3 signified a balance of supply and demand), but relatively large confidence intervals were quoted.  Prescription volumes had increased between 2001 and 2005, during which period the ADI had declined slightly. Further increases in prescription volume were estimated following the implementation of Medicare Part D. The demand-to-supply ratio for pharmacists was found to be decreasing alongside increases in prescription volume.  The multivariate analysis produced similar results to univariate analysis and indicated that no significant increase in future pharmacist workforce demand would result from Medicare Part D implementation. |
| **An overview of the main findings from the 2003 pharmacy workforce census**  The Royal Pharmaceutical Society of Great Britain’s research and development division  *The Pharmaceutical Journal* 2004, **272**:750-751  UK | Data from the 2003 pharmacy workforce census were used to produce a summary of the findings. The census excluded pharmacists with overseas addresses, pharmacists above the state pension age and pharmacists who reported in the 2002 census that they were not employed.  B3 | There was an overall response rate of 74.6%.  56% of the respondents were female.  21% of the respondent workforce was not actively participating in pharmacy.  Out of the active workforce, 72.5% held a position in community pharmacy. More than twice as many women as men work less than 33 hours per week. |
| **Overview of main census findings**  Hassell K, Shann P  The Pharmaceutical Journal 2003, **270**:314-315  UK | Data from the Pharmacy Workforce Census 2002 were processed and used to present a summary of the findings relating to pharmacists present on the register of the Royal Pharmaceutical Society of Great Britain.  B3 | There was an overall response rate of 86.2%.  Slightly over half of the respondents were female. Twice as many women as men work less than 33 hours per week.  Approximately 20% of respondents were not actively participating in pharmacy.  73% of active pharmacists work in community pharmacy and 20% work in a hospital setting. However, just under half of community pharmacists are employed in non-permanent positions. |
| **The national workforce census: (2) Older pharmacists and the pharmacy workforce in Britain**  Hassell K, Shann P  *The Pharmaceutical Journal* 2003, **270**:833-834  UK | Data from the Pharmacy Workforce Census 2002 were used to present the findings relating to pharmacists living in England, Scotland and Wales (present on the home register).  B3 | 16.6% of pharmacists on the home register were over the state pension age; of these, 35% were still active in pharmacy and 84% worked less than 33 hours per week.  A large proportion of the pharmacy workforce is also approaching retirement age; 15% are aged 50-59. |
| **The national workforce census: (3) The part-time pharmacy workforce in Britain**  Hassell K, Shann P  *The Pharmaceutical Journal* 2003, **271**:58-59  UK | Data from the Pharmacy Workforce Census 2002 were used to present the findings relating to part-time pharmacists living in England, Scotland and Wales (present on the home register).  B3 | 33% of pharmacists in Great Britain work less than 35 hours per week (part-time) and of these, 73% are female.  Part-time work patterns correlate with age and gender, but the reasons for participating in a part-time capacity are wide-ranging. |
| **The national workforce census: (4) Overseas pharmacists- does the “globalisation” of pharmacy affect workforce supply?**  Hassell K, Nichols L  *The Pharmaceutical Journal* 2003, **271**:183-185  UK | Data from the Pharmacy Workforce Census 2002 were used to present the findings relating to pharmacists living outside England, Scotland and Wales (present on the overseas register).  B3 | Overseas respondents accounted for 10.8% of pharmacists registered in Great Britain, of whom 8.4% qualified in Great Britain.  Migration of other health professionals seems to be of a similar magnitude.  Destinations of overseas pharmacists are most commonly the United States (14%), Australia (12%), Canada (11%), Hong Kong (11%) and other British destinations.  Emigration of pharmacists qualified in Great Britain is greater than immigration of overseas-qualified pharmacists.  Overseas-qualified pharmacists constitute 5.5% of the home register. |
| **The national workforce census: (5) The primary care workforce in Britain**  Mullen R, Hassell K  *The Pharmaceutical Journal* 2003, **271**:326-327  UK | Data from the Pharmacy Workforce Census 2002 were used to present the findings relating to pharmacists employed in the primary care sector living in England, Scotland and Wales (present on the home register).  B3 | Primary care pharmacists contribute 6% to the active Great British pharmacist workforce.  The majority of this group was white, female and aged 30-39.  Approximately half of all primary care pharmacists work in community pharmacy, thereby minimizing the impact that the growing primary care sector has on the employment of pharmacists in the community setting. |
| **The national workforce census: (6) The gendered nature of pharmacy employment in Britain**  Hassell K  *The Pharmaceutical Journal* 2003, **271**:550-552  UK | Data from the Pharmacy Workforce Census 2002 were used to present the findings relating to the differences between the male and female pharmacist workforce in Great Britain.  B3 | Females make up 52% of the pharmacist workforce in Great Britain.  Twice as many women work less than 33 hours per week, when compared to men.  Female respondents were slightly underrepresented in community and academic settings and disproportionately overrepresented in hospital and primary care settings.  Women also seemed to be underrepresented in hospital management positions. |
| **Career choices, working patterns and the future pharmacy workforce**  Willis S, Shann P, Hassell K  *The Pharmaceutical Journal* 2006, **277**:137-138  UK | Data were collected from a longitudinal cohort study conducted on students from 14 schools of pharmacy in Great Britain who were in their third year and would make up the 2006 pharmacy graduate cohort.  B3 | A 67% response rate was achieved.  Women were significantly more likely to be “very certain” that they would be working in hospitals in 10 years’ time, whereas the same was true for men who had entrepreneurial intentions.  There was a concern that if students failed to realize their career expectations, exit from the pharmacy workforce may result.  Only 8.1% of respondents did not expect to have a career break.  A larger proportion of students were “very certain” of practising pharmacy abroad in 10 years’ time, compared to the current proportion of the register living or working abroad (13.6% and 9.5%, respectively). |
| **Who will be tomorrow’s pharmacists and why did they study pharmacy?**  Willis S, Shann P, Hassell K  *The Pharmaceutical Journal* 2006, **277**:107-108  UK | Data were collected from a longitudinal cohort study conducted on students from 14 schools of pharmacy in Great Britain who were in their third year and would make up the 2006 pharmacy graduate cohort.  B3 | The overall response rate to the survey was 67%. The respondents were predominantly female (71.5%), which supported the growing trend of increased numbers of female pharmacy graduates.  Another pattern identified was an increase in numbers of both young pharmacists and pharmacy students who originated from ethnic minority groups.  The most compelling reason for choosing to study pharmacy at undergraduate level was because it was a science-based subject. However, caution was noted due to a possible misalignment between scientific career expectations and the social science involved in actual pharmacy practice. |
| **Graduate destinations- choices made about preregistration training**  Willis S, Shann P, Hassell K  *The Pharmaceutical Journal* 2006, **277**:164-165  UK | Data were collected from a longitudinal cohort study conducted on students from 14 schools of pharmacy in Great Britain who were in their third year and would make up the 2006 pharmacy graduate cohort.  B3 | The response rate was 67%; of these, 92.7% intended to take up pre-registration training in Britain immediately after graduation.  The proportion of preferred preregistration placements in hospital and community were approximately equal, but hospital placements were more popular among female respondents.  Students generally wanted to complete preregistration training within the region or country where they studied, which may create recruitment issues outside of these catchment areas.  Extrinsic influences primarily affected choice of preregistration posts. |
| **Workforce update – joiners, leavers, practising and non-practising pharmacists on the 2005 Register**  Hassell K, Eden M  *The Pharmaceutical Journal* 2006, **276**:40-42  UK | Data regarding the demographics of all pharmacists listed on the 2005 Register of Pharmaceutical Chemists were extracted and presented.  C1 | The total number of pharmacists on the Register decreased by approximately the same amount that it had been growing during the previous three years (2.5%). This was due to an increase in the proportion leaving the Register and not a decrease in the proportion of new entrants.  The proportion of female pharmacists continued to increase, albeit more modestly than observed in previous years.  Almost 14% joined the non-practising Register  Approximately two thirds of new entrants were female; new entrants were also more ethnically diverse, compared to the complete Register. |
| **Workforce update – joiners, leavers, practising and non-practising pharmacists on the 2006 Register**  Hassell K, Seston L  *The Pharmaceutical Journal* 2006, **277**:576-578  UK | Data regarding the demographics of all pharmacists listed on the 2006 Register of Pharmaceutical Chemists were extracted and presented.  C1 | There was a growth in the number of pharmacists on the Register of 1.4%. The proportion of female pharmacists showed an increase, compared to the previous year.  There was also a larger proportion (16%) of pharmacists present on the 2006 non-practising Register than in 2005.  The number of new entrants to the Register rose by 8% (an increasing proportion of these had overseas addresses), and in comparison to the previous year the number of pharmacists leaving the Register decreased considerably. |
| **Workforce update – joiners, leavers, practising and non-practising pharmacists on the 2007 Register**  Seston E, Hassell K, Schafheutle E  *The Pharmaceutical Journal* 2007, **279**:691-693  UK | Data regarding the demographics of all pharmacists listed on the 2007 Register of Pharmacists were extracted and presented.  C1 | The Register of Pharmacists grew by 1.9% since the previous 12 months.  The percentage of pharmacists on the non-practising Register increased from 16% in 2006 to 17.4% in 2007.  The number of pharmacists entering the Register dropped by 319 compared to the previous year.  Out of the entrants to the Register in 2007, 95.7% appeared to be “newly qualified” and the remainder were considered to be “re-entrants”.  The numbers of pharmacists leaving the Register decreased compared to the previous year and interestingly, roughly equal proportions of men and women left the register, whereas in the past men were significantly more likely to leave than women.  There were 142 independent prescribers and 1257 supplementary prescribers on the Register. |
| **Destination, future intentions and views on practice of British-based pharmacists 5 and 10 years after qualifying**  Hassell K  *Pharmacy World of Science* 2006, **28**(3):116-122  UK | A postal questionnaire was sent out to registered pharmacists with addresses in Great Britain in 2003. Overseas pharmacists and pharmacists who were over state pension age or not working at the time of the previous census were omitted.  Data relating to two specific cohorts were analysed and compared: those who had been on the Register for five years (qualified in 1997/8) and 10 years (qualified in 1992/3).  B3 | The response rates received from the 1993 and 1998 qualification cohorts was 62% and 58%, respectively.  The economic activity of a notable proportion of female pharmacists decreases relatively shortly after qualifying, with 13% of the 1993 and 8% of the 1998 qualified respondents being economically inactive in 2003; 41% of females with 10 years’ experience worked part-time, compared with 14% of those with 5 years’ experience in 2003.  Pharmacists with 10 years’ experience were more likely to work in primary care and younger pharmacists were more likely to work abroad.  Pharmacists working in a community setting were more likely to have a weak desire to practise pharmacy or regret becoming a pharmacist than those working in either hospital or primary care sectors. |
| **A pharmacy study in the West Midlands: (1) Current work profiles and patterns**  Blenkinsopp A, Boardman H, Jesson J, Wilson K  *The Pharmaceutical Journal* 1999, **263**:909-913  UK | A questionnaire was designed and posted to all pharmacists up to the age of 65 (and those over 65 years who were still working in pharmacy) within the West Midlands region.  B3 | The response rate was 68.8%; 90% of the respondents were actively working in pharmacy; 53.1% of respondents were female. Pharmacists in non-white ethnic groups were overrepresented in community and underrepresented in hospital sector employment.  The majority of respondents worked between 31 and 40 hours per week.  One third of locum pharmacists reported working over 30 hours per week.  Slightly more than a quarter of respondents described working part-time; the vast majority of these were female. |
| **A pharmacy study in the West Midlands: (2) Changes made and planned for the future**  Boardman H, Blenkinsopp A, Jesson J, Wilson K  *The Pharmaceutical Journal* 2000, **264**:105-108  UK | A questionnaire was designed and posted to all pharmacists up to the age of 65 (and those over 65 years who were still working in pharmacy) within the West Midlands region.  B3 | The response rate achieved was 68.8%.  The level of inter-sector mobility between hospital and community settings was small, with the majority of pharmacists changing sector being under age 30.  Almost a quarter of respondents had reduced their hours.  The most common reason reported for changes made to working habits in the last three years was “seeking more interesting work/greater job satisfaction”.  14.4% of the respondents exhibited a preference to change sector of employment and 18.8% reported a desire to leave the profession to pursue another career; in both cases, non-white pharmacists were overrepresented. |
| **A pharmacy workforce study in the West Midlands: (4) Morale and motivation***  Boardman H, Blenkinsopp A, Jesson J, Wilson K  *The Pharmaceutical Journal* 2001, **267**:685-690  UK | A questionnaire was designed and posted to all pharmacists up to the age of 65 (and those over 65 years who were still working in pharmacy) within the West Midlands region. A purposive sample of 80 of the questionnaire respondents were contacted to take part in a semistructured telephone interviews.  B3 | The response rate obtained for the questionnaire was 68.8%.  Pharmacy was revealed as the first choice of career for approximately two thirds of respondents; 46.1% described their career as satisfying. Interestingly, pharmacists working in the community sector were the least satisfied with their careers, compared to pharmacists in all other settings.  One third of respondents were satisfied with their working conditions and a similar proportion didn’t feel challenged enough by their work.  The majority of pharmacists wanted to work more closely with other health care professionals (60.4%).  Approximately half of the respondents (47.3%) were apathetic towards the future of the profession. |
| **Does community pharmacy offer women family-friendly working conditions and equal opportunities? The accounts of female community pharmacists over the age of 30**  Gidman WK, Hassell K, Day J, Payne K  *International Journal of Pharmacy Practice* 2007, **15**:53-59  UK | 30 face-to-face, semistructured interviews were conducted with female pharmacists. These pharmacists were, female, over the age of 30 and living in north-west England.  242 recruitment letters and consent forms were sent to those meeting the sample requirements; a reminder was sent to non-responders.  Interview transcripts were analysed until thematic saturation was reached.  B3 | A response rate of 40% was achieved for the consent forms; from this, a purposive sample of 30 respondents were interviewed.  Approximately two thirds of interviewees acted as carers for other family members (n=21) and worked part-time (n=19).  Many interviewees reported changing their employment patterns due to a change in marital status and family responsibilities.  Intrinsic factors such as “age and number of children, availability of informal childcare, views on formal childcare provision, husband’s job and contribution to domestic workload” were very important in determining working patterns.  Extrinsic factors such as “inflexible working conditions, limited availability of family-friendly contracts, antisocial working hours, long hours, allocation of annual leave, supervisory requirements, remuneration, high-pressure working environment and incompatibility of pharmacy working with formal childcare” were important influences in female work patterns.  Discriminatory employment practices were not perceived to be a problem.  Employers and policy-makers were recommended to reduce extrinsic barriers to female participation in the workforce. |
| **The implications of increasing student numbers for pharmacy education***†  Taylor KMG, Bates IP, Harding G  *Pharmacy Education* March 2004; **4**(1):33-39  UK | Discussion of relevant literature relating to the issues surrounding increasing pharmacy student enrolments.  D | There was an increase in the overall number of students attending university from the 1970s to 2000. This increase in numbers was mirrored in pharmacy school intake levels; however there was not a corresponding increase in resources.  The demand for pharmacists remained higher than the supply of newly qualified pharmacists, despite increased student numbers.  New schools of pharmacy were planned to open.  The academic standard of applicants was presumed to decrease, which may lead to a ”dumbing down” of the course.  Computer technology had increased in importance in delivering the course.  The number of pharmacy-trained academic staff had declined.  The number of preregistration places did not increase in line with the increased number of pharmacy graduates.  The implementation of a two-tier pharmacy education was perceived. |
| **Pharmacy in Developing Countries***  Felicity Smith. In: *Pharmacy Practice*. Edited by Taylor K, Harding G. London: Taylor & Francis; 2001:95-96  UK | A summary of pharmacy education and services in developing countries.  D | Egypt was recognized as a country that exports trained health care workers, such as pharmacists, to the Middle East, USA, Canada and Western Europe.  Pharmacy education varied from country to country to adapt to the health care needs of the local population. This was evidenced in China, where elements of allopathic and traditional Chinese medicine were taught alongside each other.  Universities were predominantly located in urban areas and as such students generally had limited contact, understanding and skills to address health problems in rural areas. |
| **Snapshot of the Australian Public Hospital Pharmacy Workforce in 2005***  O’Leary KM, Allinson YM  *Journal of Pharmacy Practice and Research* 2006, **36**(2):103-106  Australia | A questionnaire was sent to 198 public hospitals identified as having at least 50 beds and providing hospital pharmacy services. This was followed up by a reminder letter to non-respondents.  B3 | Responses were received from 99 out of 198 hospitals, but this varied across hospital type and location.  There was a decrease in the number of full-time equivalent pharmacist vacancies in 2003 to 7%, but this may be attributed in part to a decrease in the number of positions or to non-response bias.  There was a slight reduction in the number of pharmacists working part-time (31% in 2005 and 34% in 2003), and a decline in the proportion of pharmacists with postgraduate qualifications (28% in 2005 and 30% in 2003).  Despite an increase in pharmacy graduates, there was no corresponding increase in the number of preregistration positions available in public hospitals.  The proportion of pharmacy technicians with formal qualifications increased from approximately 38% in 2003 to 45% in 2005.  There was a decline in the numbers of pharmacy technicians and relevant support staff in every type of hospital. This may have been due to an increased use of technology, improved efficiency and the introduction of the Pharmaceutical Benefits Scheme. |
| **Snapshot of the Australian public hospital pharmacy workforce in 2003***  O’Leary KM, Allinson YM  *Journal of Pharmacy Practice and Research*  2004, **34**(2):104-108  Australia | A questionnaire was sent to 303 hospitals with hospital pharmacy services (239 public hospitals and 64 private hospitals). The response rate from private hospitals was too low to be used in the analysis, thus all data analysed related to the 128 public hospital pharmacy service providers that responded.  B3 | An overall response rate of 54% was obtained, but this varied across hospital classification and location.  There was a reduction in the number of vacant full-time equivalent pharmacist positions from 2001 (14% reduced to 10%).  Approximately one in three pharmacists worked part-time and a similar proportion held postgraduate qualifications.  The ratio of preregistrant positions to full-time equivalent pharmacists was higher in 2003 than in 2001 (1 to 7.8 and 1 to 10.2, respectively).  More than 60% of pharmacy technicians did not have any formal qualifications.  Important recruitment strategies included: increasing the exposure of pharmacy students to hospital pharmacy during training and registration; accelerated pay within the hospital pharmacy award; using the Internet to advertise vacancies; and the employment of overseas pharmacists. |
| **Snapshot of hospital pharmacy workforce in Australia**  O’Leary KM, Allinson YM, Kirsa SW, Jackson JK, Burgess NG  *Journal of Pharmacy Practice and Research* 2002, **32**(1):72-76  Australia | Hospital pharmacy services were identified in 279 of more than 1000 registered hospitals in Australia. A questionnaire was sent to 248 hospital pharmacy services (contact details were unavailable for 31 pharmacy services).  B3 | 101 questionnaires were received, which represented a response rate of 41% of those surveyed and 36% of all hospital pharmacy services nationwide. Responses varied by hospital type and location.  Vacancies for full-time equivalent pharmacists represented 14% of establishment positions.  An average of 32% of hospital pharmacists worked part-time and as such, 1.2 pharmacists were required to cover every 1 full-time equivalent pharmacist.  The respondents revealed that one third of all hospital pharmacists had postgraduate qualifications.  The ratio of preregistrant positions to full-time equivalent pharmacists was 1 to 10.2.  The surveyed respondents reported that pharmacists’ time was divided so that 41% was spent on clinical services, 39% on distribution services and 16% on management services. Interestingly, 9% of support staff also performed management activities. |
| **Factors affecting the recruitment and retention of pharmacists to practice sites in rural and remote areas of New South Wales: A qualitative study**  Harding A, Whitehead P, Aslani P, Chen T  *Australian Journal of Rural Health* 2006, **14**:214-218  Australia | Qualitative semistructured in-depth interviews conducted on a purposive sample of 12 out of 13 contacted community pharmacists in rural or remote areas of New South Wales.  B3 | The majority of respondents regarded that having “previously lived in rural areas, availability of better business opportunities, and economic reasons” were the principal motivators for rural recruitment.  Retention factors identified for rural pharmacists included: job satisfaction resulting from an extended role; close relationships with customers and other healthcare professionals; and rural customers’ increased willingness to accept advice. Factors that negatively affect recruitment were high workload and the limited availability of locums and face-to-face continuing professional education.  The shortage of rural and remote pharmacists was attributed to the increasing proportion of females in the workforce and the difficulty of finding their partner employment, negative preconceived ideas of rural life and pharmacy practice and the rural shortage of doctors. |
| **The first graduate cohort at Charles Sturt University: What Impact on the rural pharmacist shortage?***  Simpson MD, Wilkinson JM  *Journal of Pharmacy Practice and Research* 2002, **32**:69-71  Australia | A descriptive study based on data collected from a questionnaire completed in 1999 by pharmacy students in their second and third years at Charles Sturt University and an abbreviated version of the questionnaire filled in by final-year students in 2000.  In 1999, 69 out of 93 second and third years completed the full questionnaire and in 2000 all the fourth year students (37) completed the shortened questionnaire.  C1 | In 1999 the questionnaire results revealed that 65% of respondents wanted a career in community pharmacy, whereas 3%-6% were interested in careers in research, industry or hospital and 22% were unsure as to their career preference. The majority of students favoured a career in a rural location (56%), approximately a quarter of students wanted to work in metropolitan areas, no respondent reported wanting to work in a remote area, and 20% were undecided regarding their future career location.  The primary reason that influenced the choice of career location was lifestyle (45%).  Rural areas accounted for 62% of the confirmed preregistration location for final year students and 38% obtained placements in metropolitan areas. |
| **Workforce and service delivery analysis across New Zealand hospital pharmacy departments***  Plant EA, Norris PT, Tordoff JM  *Journal of Pharmacy Practice and Research* 2006, **36**(4):271-274  New Zealand | A questionnaire was designed and sent to the chief pharmacist in all 30 public hospitals that employed at least one pharmacist. It was administered in November 2001 and again in November 2003 (with an additional question relating to salaries).  B3 | The response rate was 83% for both surveys.  The number of funded FTE pharmacists increased between 2001 and 2003.  Vacancies for pharmacists decreased from 24% to 15% and vacancies for technicians increased from 10% to 14% between 2001 and 2003.  The majority of pharmacists and pharmacy technicians were female in 2003 (78% and 95%. respectively).  Key workforce issues identified were: difficulties recruiting/retaining suitably experienced staff; poor salaries compared to community sector; poor funding; high staff turnover; large number of young pharmacists leaving to work abroad; high dependency on temporary overseas pharmacists; high proportion of females.  Also in 2003 it was noted that higher wages for hospital pharmacists existed in Australia, there was a lack of structured career path for technicians and there were high expectations for service delivery but limited funding available. |
| **Pharmacy manpower in Lebanon: An exploratory look at work-related satisfaction***  Salameh P, Hamdan I  *Research in Social and Administrative Pharmacy* 2007, **3**(3):336-350  Lebanon | Cross-sectional descriptive study of registered Lebanese pharmacists actively practising in Beirut and the surrounding suburbs.  A systematic random sample was selected in which every second name was chosen as a potential participant. These participants were then interviewed and the resulting data were analysed by means of SPSS software, version 12.0 (SPSS Inc., Chicago, IL)  B3 | Out of the 243 selected pharmacists, 229 participated.  There were more female than male participants and more single than married pharmacists.  Recently graduated pharmacists were present in higher proportions in community and medical representation settings.  A small proportion of pharmacists (20%) experienced difficulties in obtaining employment, but the vast majority of interviewees (96.1%) believed there was “a lack of opportunities in the pharmaceutical sector”. There was a belief that the employment market was saturated.  Pharmacists with additional postgraduate qualifications appeared to be employed as pharmacists for longer than those without additional qualifications.  Approximately half of the respondents had remained in their initial job, especially academic pharmacists, none of whom had changed their career orientation.  Pharmacy education curricula were not unified; there were doctorate and bachelor of science degree programmes allowing entry to the same work settings. Curricula were not always aligned with employment activities, especially seen in clinical pharmacy, which was taught in some universities but has not yet been implemented in Lebanese hospitals. |
| **Effect of pharmacist emigration on pharmaceutical services in southern Africa**†*  Katerere DR, Matowe L  *American Journal of Health-System Pharmacy* 2003, **60**:1169-1170  USA | A descriptive journal article providing information about the extent and affect of pharmacist migration in southern Africa.  D | Approximately 1000 pharmacy students graduate each year in South Africa and about 40 pharmacists a year qualify in Zimbabwe.  In 2001 alone, 600 pharmacists registered in South Africa and 60 pharmacists in Zimbabwe emigrated.  Botswana invested in sponsoring students to train in pharmacy abroad, due to having no schools of pharmacy in the country, but few of these students return.  Unlike physician consultations, pharmacist advice was delivered free of charge.  Public sector and academic pharmacists were in great need.  The exchange of professional expertise was favoured, as opposed to limiting the free movement of pharmacists. |
| **Migration of health professionals in six countries: a synthesis report***  Awases M, Gbary A, Nyoni J, Chatora R  World Health Organization 2004  Available at: [<http://www.hrhresourcecenter.org/node/61>]. Accessed 22.03.08  Africa | The study was conducted in Cameroon, Senegal, Ghana, South Africa, Uganda and Zimbabwe.  Data were collected using eight different methods:   - questionnaire for Country Survey on Secondary Data on Trends of Migration; - interviews with professional informants in the health system; - questionnaire for individual health workers; - focus group discussions with key community stakeholders; - interviews with key informants in the community - questionnaire for refugees/migrants living outside their home countries - questionnaire for returnees - interviews with internal migrants, viz. professionals who had moved to the private sector.   B3 | The number of health professionals had increased in all six countries studied from 1991 to 2000, but the increase in the number of pharmacists was less prominent than those observed among dentists and doctors.  From 1991 to 2000 the number of pharmacists registered in Cameroon increased by 79%, in Senegal an increase of 52% was reported and an increase of 8% was experienced in South Africa.  There was a marked decrease in the proportion of pharmacists employed in the public sector in South Africa (decrease of 41% from 1998 to 2002), and in Uganda (decreased from 167 pharmacists in 1993 to 37 in 2000), whereas in Ghana the decrease was marginal. Nevertheless, there was a large increase in the number of pharmacists working in the public sector in two francophone countries: the proportion in Cameroon increased by 71% between 1991 and 2000, and by 83% for Senegal between 1993 and 2000. In addition, the number of pharmacists working in the private sector increased in Cameroon from 50 in 1991 to 90 in 2000 and increased in Senegal from 210 in 1993 to 306 in 2000.  The total number of registered pharmacists in Cameroon expanded rapidly during the 1990s.  Senegal experienced a fall in the number of pharmacists in the public sector from 1993 to1996, to a low of 5%, but this improved to 18% in 2000.  In South Africa the total number of registered pharmacists increased, but the number working in the public sector decreased from 22% of total pharmacists in 1998 to 12% in 2001.  Almost all pharmacists in Uganda work in the public sector.  No data on the registration of pharmacists were available from 1991 to 2000 in Zimbabwe.  The number of pharmacists trained in South Africa and Ghana increased over the period studied (increases of 167% and 35% respectively). Ghana didn’t produce any pharmacists in 1995, due to industrial action taken by academic staff.  Health care professionals in different countries and professions cited many migration factors; each had varying levels of importance. |
