# Supplementary material for: The global pharmacy workforce: a systematic review of the literature
Source: Hum Resour Health. 2009 Jun 19;7:48. doi: 10.1186/1478-4491-7-48 (PMC2706790; doi:10.1186/1478-4491-7-48)
Supplement: Additional file 2 — Table 2. Record of non-peer reviewed evidence. Annotated references. [file 1478-4491-7-48-S2.doc]

**Table 2. Record of non-peer reviewed evidence**

* Denotes that funding was not explicitly declared

† Denotes that no methodology was described

| **Reference** | **Methodology, research design & evidence category** | **Key findings** |
| --- | --- | --- |
| **The Pharmacist Workforce: A Study of the Supply and Demand for Pharmacists*** (December 2000) Department of Health and Human Services, Health Resources and Services Administration and Bureau of Health Professions  Available from:  <http://bhpr.hrsa.gov/healthworkforce/reports/pharmacist.htm>  (accessed 18.03.08)  US | Report to congress conducted by the Health Resources and Services Administration. Data source included published articles from peer-reviewed journals, reports from academic, governmental and private research groups and analyses and reports conducted or commissioned by professional associations and pharmacists.  C1 | Pharmacists are the third largest group of healthcare professionals in the United States.  In the year 2000 there were approximately 196,000 active pharmacists in the US.  A shortage of pharmacists existed which was primarily due to an increase in demand together with a limited supply of pharmacists.  Demand factors such as prescriptions, administrative volume and complexity of medication therapy all increased.  There were increased vacancy rates (especially in the federal sector) coupled with difficulties hiring pharmacists and an increasing workload.  The number of active pharmacists was expected to grow from 196,000 in 2000 to 224,500 in 2010. |
| **National Pharmacist Workforce Survey: 2000**  Pedersen CA, Doucette WR, Gaither CA, Mott DA, Schommer JC  Pharmacy Manpower Project, August 2000  Available at:  <http://www.aacp.org/site/page.asp?TRACKID=&VID=1&CID=503&DID=3897>  (accessed 05.03.2008)  US | A questionnaire was designed and delivered to a systematic sample of 5000 pharmacists, 4,895 were presumed to have received the survey. Non-respondents were contacted up to a maximum of 5 times.  B3 | The response rate was 46%.  88.2% of respondent pharmacists were actively participating in pharmacy  44% of active pharmacists were female. The average age of male full time pharmacists was 46.2 whereas females averaged 37.4 years.  The full time pharmacists worked an average of 44.2 hours per week. Second jobs were more prevalent in those working part-time 18% than full-time 12.3% |
| **National Pharmacist Workforce Survey: 2004**  Mott DA, Doucette WR Gaither CA, Kreling DH, Pedersen CA and Schommer JC.  Pharmacy Manpower Project, March 2006  Available at:  <http://www.aacp.org/Docs/MainNavigation/Resources/7295_final-fullworkforcereport.pdf>  (accessed 05.03.2008)  US | A random sample of 6,000 pharmacists was obtained these were allocated at random into six groups of 1,000; one group were a hold-back sample, another group received a core survey and three groups received the core survey and one of three supplemental surveys. The final group received the core survey and composite supplemental survey. Non-respondents were contacted up to a maximum of 5 times.  B3 | The response rate was 33.8%.  86% of respondent pharmacists were actively participating in pharmacy, of these, 45.9% were female. The average number of hours worked per week by full time pharmacists was 43.4. The proportion of pharmacists working with 3 or more technicians increased between 2000 (33%) and 2004 (46%). There was an increase in the proportion of pharmacists personally dispensing more than 160 prescriptions daily; 23% in 2000 and 36% in 2004. Pharmacists generally were more positive towards work in 2004 and turnover intention decreased in all sectors except supermarkets. |
| **Workforce Trends of Pharmacists for Selected Provinces and Territories in Canada**  Canadian Institute for Health Information  2006 (Ottawa: CIHI, 2007)  Available from:  <http://www.icis.ca/cihiweb/dispPage.jsp?cw_page=hhr_pharm_e>  (accessed 18.03.08)  Canada | Includes information from submissions of annual registration forms from actively registering pharmacists as of October 1 2006. Pharmacists applying for inactive registrations were excluded and data from Manitoba, New Brunswick, Newfoundland  and Labrador and Quebec and from the territory of Nunavut were not available.  B3 | It was revealed that 87.7% of the pharmacy workforce was actively employed in pharmacy.  The distribution of pharmacists between urban, rural and remote settings varied greatly across the provinces and territories; the largest proportion of pharmacists in urban settings were in Ontario and British Columbia (91.6% and 89.9% respectively) whereas the largest proportion of pharmacists in rural areas was on Prince Edward Island (19.1%) and the largest percentage in remote areas were in Saskatchewan (21.2%).  The percentage of female workforce was greater than male in all the territories/provinces analysed, except in the Northwest Territories where their numbers were equal.  The age distribution across the province/territories varied slightly with a higher proportion of pharmacists aged 20-29 years in the Northwest Territories.  At least 2/3rds of all new graduates in Ontario, Alberta, British Columbia and Saskatchewan were female.  The majority of pharmacists had a single employer and a permanent position.  Approximately one third of pharmacists were pharmacy managers in their primary employment position.  Community pharmacists account for over 75% of the workforce in all selected provinces. |
| **Hospital Pharmacy in Canada – 2005/06 Annual Report**  Wilgosh C, Hall KW, Babich M, Johnson N *et al.*  2007  Personal communication  Canada | A list of 203 hospital pharmacies was collated; 193 of these met the inclusion criteria which included hospitals with more than 50 acute beds and more than a total of 100 beds. Mental health facilities were excluded.  An online questionnaire was designed and Directors of Pharmacy were provided with details of how to log on to the survey website.  Resultant data from questionnaires were only included in analysis if more than 25% of key questions were answered.  Non-responders received weekly email reminders.  B3 | The response rate achieved was 74%.  Key findings relating to pharmacy human resources:  The number of management, pharmacist and pharmacy technician positions increased since the previous survey.  The skill mix of the pharmacy workforce and the proportion of pharmacists’ time spent on various activities hadn’t altered much compared to earlier years.  The annual average growth in salary for FTE pharmacy staff had decreased compared to previous years.  Pharmacist vacancies were detailed by 73% of respondents, a pharmacist vacancy rate of 13.3% was reported and a reduction in the duration of vacancies decreased to 182 days on average.  The technician vacancy rate increased to 2.1%.  11.8% of all pharmacists, 16.2% of management staff and 8.4% of pharmacy technicians were expected to retire within 5 years. |
| **The Pharmacy Technician Workforce in Canada: Roles, Demographics and Attitudes**  **Part I – Responses to National Survey of Pharmacy Technicians and Assistants**  **Part II – Responses to National Survey of Pharmacists (Owners and Managers)**  Vision Research  March 2007  Available at:  <http://www.pharmacyhr.ca/ResearchAndReports.aspx>  (accessed 18.03.08)  Canada | A representative sample of 3000 pharmacists were contacted directly and asked to direct the most senior manager or owner to complete a questionnaire designed for them and to pass on another questionnaire to pharmacy technicians on their staff.  The findings from the two different questionnaires were interpreted and reported on separately.  The response rate varied regionally.  B3 | **Part I – Responses to National Survey of Pharmacy Technicians and Assistants:**  2087 completed questionnaires were received from pharmacy technicians.  A massive majority of technician respondents were female (94%).  Similar proportions of technicians worked in community settings (53%) as in hospital or long-term care facilities (44%).  Only 13% of the pharmacy technician workforce reported working less than 30 hours per week.  Nearly two thirds of technicians reported intending to remain in the workforce for at least 10 years.  The level of education and training of technicians varied greatly.  1.13 full-time equivalent (FTE) technicians per pharmacist were reported in community settings, whereas 1.32 FTE technicians per pharmacist were reported in hospital settings.  Generally job satisfaction among technicians was high except relating to opportunities for career development.  84% supported national accreditation of community college programmes and 73% supported mandatory completion of one of these courses.  Voluntary certification was supported by 70% of technicians whereas mandatory certification was supported by 62%.  At least 84% were somewhat interested in becoming a regulated pharmacy technician.  **Part II – Responses to National Survey of Pharmacists (Owners and Managers):**  973 completed pharmacist questionnaires were received.  A ratio of 1 technician FTE per pharmacist FTE in community was reported, and a ratio of 1.1 technician FTEs per pharmacist FTE was reported in hospital settings. These ratios were lower than expected.  Hospital respondents were twice as likely to have college-educated or certified technicians on their staff as community respondents.  Similar proportions of pharmacists and technicians supported national accreditation of technician training programmes (86% and 84% respectively).  65% of pharmacists supported either voluntary or mandatory certification.  More experienced pharmacists were generally more willing to support technician’s expanded roles and responsibilities. |
| **An Environmental Scan of Pharmacy Technicians (Roles and responsibilities, education and accreditation, and certification)**  September 2006  Blackburn J, Blackburn and Associates  Available from:  <http://www.pharmacists.ca/content/about_cpha/whats_happening/cpha_in_action/pdf/PharmacyTechniciansEnvironmentalScan.pdf>  (accessed 21.03.08)  Canada | A descriptive report on the demographics, roles and responsibilities and capacity for advancement of pharmacy technicians on a provincial, national and international level.  D | Many national organisations have supported and developed policies for expanding the roles of pharmacy technicians, national accreditation of technician courses, regulation, certification, examination and registration.  In Alberta pharmacy technicians who passed a voluntary examination became certified.  In Ontario technicians may also become certified.  Québec had an official category of Assistant-Technique en Pharmacie who had obtained a diploma from a pharmacy assistance course and were allowed to complete a list of delegated tasks.  In Australia there were various levels of certification (I-IV) via completion of nationally accredited courses.  In Denmark ‘pharmaconomists’ are qualify after successfully completing a three year course after which they may dispense prescriptions, provide advice on drugs and sell drugs independently of a pharmacist.  In New Zealand, pharmacy technicians must hold or be studying for an approved certificate issued by the Pharmaceutical Society of New Zealand which enables them to compound and dispense medication under pharmacist supervision.  In the UK voluntary registration of pharmacy technicians with the Royal Pharmaceutical Society of Great Britain was available.  In the United States a national, voluntary certification programme was developed through the Pharmacy Technician Certification Board. |
| **Environmental Scan of Pharmacy Technicians** *  September 2001  MacInnis M, Power B, Cooper J  Available at:  <http://www.pharmacists.ca/content/hcp/resource_centre/practice_resources/pdf/pharmacy_technicians.pdf>  (accessed 21.03.08)  Canada | A report on the roles, responsibilities, training and certification of pharmacy technicians nationally, provincially and internationally.  D | There was no national body responsible for accrediting pharmacy technician-training programmes.  It was recognised that technicians required more specialised skills to work in hospital settings and that their roles were more defined than community pharmacy technicians.  Ontario and Alberta were the only two provinces to have developed voluntary certification programmes.  The United States had a national, voluntary certification exam and accredited pharmacy technician-training programmes.  The United Kingdom had nationally recognised courses for medicines counter assistants, dispensing assistants and dispensing technicians.  Australia had a four-level, nationally recognised, accredited training programme for pharmacy assistants.  In New Zealand a pharmacy technician must hold a certificate from the Pharmaceutical Society of New Zealand and a National Certificate in Pharmacy (Technician). |
| **A Situational Analysis of Human Resource Issues in the Pharmacy Profession in Canada**  May 2001  Prepared by Peartree Solutions Inc.  Available at:  <http://www.pharmacyhr.ca/Articles/Eng/68.pdf>  (accessed 18.03.08)  Canada | Review of the relevant literature on pharmacist workforce issues in Canada. Data included was from relevant literature, internet sites and public databases. Twenty three pharmacists were also interviewed to gather their views on issues affecting the pharmacy workforce.  B3 | Nearly 80% of Canada’s pharmacists worked in the community sector.  Traditional signs of a labour shortage in pharmacy were evident such as, low rates of unemployment, increased numbers of vacancies which took longer times to fill, increased overtime and increased wages in excess of the cost of living.  It appeared that there was increased difficulty in recruitment and retention of pharmacists in rural areas.  Increasing demand for pharmacists was thought to be due to several professional and business related factors. Supply factors were also explored.  The average retirement age in Canada had dropped below the age of sixty.  A key barrier to the expansion of pharmacists’ roles beyond dispensing services was the lack of compensation.  There was no available evidence regarding the cause of the pharmacist shortage or the predictable future demand and supply issues.  Data available were often “inconsistent, incomplete and, therefore [an] incoherent picture of the labour market for pharmacists”.  More data were required to prepare a model for the future demand and supply of pharmacists. |
| **Blueprint for Pharmacy revised draft 5.6**†*  February 2008  Personal communication  Canada | Development of a strategic plan for the future pharmacy profession in Canada. Draft based on pharmacy consultation, no methodologies detailed.  D | Five strategic areas requiring action were identified to fulfil future advancement of the pharmacy profession in Canada these included; pharmacy human resources, education and continuing professional development, information and communication technology (ICT), financial viability and sustainability and legislation, regulation, and liability.  With regards to pharmacy human resources the strategy included; gaining a better understanding of job satisfaction of pharmacists and pharmacy technicians, supporting international pharmacy graduates, promotion of best practices to improve efficiency of the workforce and increasing the level of qualification, responsibility and accountability of pharmacy technicians. |
| **Pharmacy workforce census 2005: Main findings**  Hassell K, Seston L, Eden M  August 2006  Available at:  <http://www.rpsgb.org.uk/pdfs/census05.pdf>  (accessed 07.03.2008)  UK | A comparative analysis was conducted on all registered pharmacists using data extracted from the Register on 6^th^ August 2004 and 5^th^ August 2005.  The second component of the census was a postal questionnaire sent out to registered pharmacists with addresses in Great Britain. Overseas pharmacists were excluded from the census.  B3 | There was a slight decline in membership numbers between 2004 and 2005 which was largely attributed to the introduction of a two part register of practising and non-practising pharmacists. Notably there was an increase of 300 entrants in 2005 compared to 2004.  As observed in previous years, the 30 to 39 age bracket accounts for the majority of pharmacists.  The response rate to the mailed survey was 76.6%.  Since the survey in 2003 there was an increase in the participation of pharmacists in 2005 (78.8% and 81.2% respectively).  14% of registered pharmacists were recorded on the non-practising Register.  The proportion of pharmacists employed in the community sector has decreased since 2003 by approximately 2% but was still the sector employing the majority of pharmacists (70.1%).  The average hours worked per week for active pharmacists in Great Britain was 35 hours. This represented an increase of three hours from 2003.  There was a slight decrease in the proportion of pharmacists working part-time compared to 2003.  Generally high levels of job satisfaction were reported. |
| **Pharmacy workforce census 2003: Main findings**  Hassell K  July 2004  Available at:  <http://www.rpsgb.org/pdfs/census0309.pdf>  (accessed 07.03.2008)  UK | A comparative analysis was conducted on all registered pharmacists using data extracted from the Register on 10^th^ August 2002 and 8^th^ August 2003.  The second component of the census was a postal questionnaire sent out to registered pharmacists with addresses in Great Britain. Overseas pharmacists and pharmacists who were over state pension age or not working at the time of the previous census were omitted.  B3 | The Register exhibited a growth of 2.4% from the previous year (equivalent to 1118 pharmacists). The pharmacist workforce consisted of 52.2% female members.  Similarly to last year the largest proportion of pharmacists was aged between 30 and 39 years, although the less than 29 age group saw the largest increase in number, possibly due to increased student numbers.  The questionnaire elicited a response rate of 74.6%.  The proportion of pharmacists actively contributing to pharmacy declined from 2002 to 79%.  The community sector was still the largest employer of pharmacists. However the hospital and primary care sectors had increased the number of employment opportunities for pharmacists and as a result the number of pharmacists employed within these sectors increased over the year studied.  There was low cross-sector mobility observed when compared with 2002 data.  There was a decrease in the average number of hours worked per week in the active pharmacy profession during 2002-2003 (35 and 32 hours per week respectively).  The desire to practice pharmacy was generally “strong”, however this seemed to decrease with advancing age. |
| **Pharmacy Workforce Census***  Hassell K, Shann P  February 2003  Available at:  <http://www.rpsgb.org/pdfs/census03.pdf>  (accessed 07.03.2008)  UK | All pharmacists registered with the Royal Pharmaceutical Society in August 2002 were requested to complete a postal survey.  B3 | A high response rate of 86.2% was obtained. 52.6% of the respondents were female. The largest proportion of pharmacists were aged 30-39 years. Approximately 20% of the workforce were not actively participating in pharmacy. Of those in active pharmacy employment 73% work in the community, 20% in hospital, 6% in primary care and 12% in other settings (some respondents had more than one job). Almost two thirds of primary care pharmacists have two or more jobs.  The majority of pharmacists (39.6%) work between 33 and 40 hours per week.  More than double the proportions of females work less than 33 hours per week compared to males (40.3% and 19.4% respectively). |
| **Future Pharmacy Workforce Requirements; Workforce Modelling and Policy Recommendations Executive Report**  Guest D, Battersby S, Oakley P  March 2005  Available at:  <http://www.rpsgb.org/pdfs/futphwfreqexecrept.pdf>  (accessed 10.03.2008)  UK | The report is based on data collected through a many different techniques, these included; an analysis of published and unpublished literature, interviews with a sample of representatives from different pharmaceutical sectors, a Delphi exercise among the Advisory Group and attitude surveys among samples of pharmacists and pharmacy technicians.  B3 | A dynamic pharmacy workforce model based on predefined assumptions and demand/supply factors was established.  There were three key issues highlighted that increased cross-sector demand for pharmacists; “Professional Quality Assurance”, “Organisation of Pharmacy Provision” and “Healthcare Expansion”.  Sector-specific assumptions of factors affecting the supply and demand of pharmacists were listed.  Projections of the demand and supply of pharmacists revealed a deficit of 16,752 by 2013.  Recommendations were made to alter the supply and demand assumptions that the workforce model was based on in order to cause convergence of demand and supply. |
| **Supply Of and Demand For The Pharmacy Workforce In Great Britain**  Hassell K  May 2003 Available at:  <http://www.pharmacy.manchester.ac.uk/cpws/publications/Reports/supply.pdf>  (accessed 13.03.2008)  UK | This report was used to inform decisions regarding recommendations to end the control of entry regulations for community pharmacies in the UK.  Data were obtained from several sources, these included; The Pharmacy Workforce Census conducted in 2002, Universities and Colleges Admissions Service, the Royal Pharmaceutical Society of Great Britain, The Pharmaceutical Journal and the Office of Fair Trading report titled The control of entry regulations and retail pharmacy services in the UK, 2003.  C1 | The proportion of inactive pharmacists in the UK pharmacy workforce was 30%, consisting of pharmacists who lived or worked overseas, unemployed pharmacists and those registered pharmacists who work outside pharmacy.  Approximately 14% of “inactive” pharmacists and those working outside pharmacy thought they would return to pharmacy within 12 months.  The rate of entry of overseas pharmacists increased in 2001 and 2002 compared to previous years.  The numbers of pharmacists trained in Great Britain leaving the UK to work overseas exceeded the numbers of overseas pharmacists joining the Register.  There appeared to be a shortage of pharmacists to meet the pre-existing level of demand in community pharmacy, if demand was added to by removal of the entry regulations this may worsen the shortage. |
| **Comprehensive Review of the Pharmacy Workforce**  KPMG Consulting for the Department of Health, Social Services and Public Safety.  May 2001  Available at:  <http://www.dhsspsni.gov.uk/wfp_comprehensive_review_of_the_pharmacy_workforce.pdf>  (accessed 10.03.08)  UK | This review of the pharmacy workforce was conducted in Northern Ireland. Data was collected from a variety of sources including; a literature review and desk research, twenty semi-structured face-to-face key informant interviews, focus groups included forty five participants and a Pharmacist Workforce Telephone Survey which consisted of a structured questionnaire was administered to a cross-section of 500 pharmacists in Northern Ireland during the period of February to April 2001.  B3 | The Pharmaceutical Society of Northern Ireland (PSNI) had 1699 registered pharmacists with male and females represented in equal proportions. 69% of these were actively practising pharmacy. 109 pharmacists joined the PSNI register while 105 pharmacists left the register in the previous 12 months.  Out of the survey respondents the majority of active pharmacists worked full-time (78%) and in the community (81%) where 58% reported working in an independent pharmacy.  21% of respondents reported pharmacist vacancies at their workplace (with a larger proportion for hospital pharmacists).  Almost one fifth (18%) of respondents reported intentions of leaving active pharmacy either for a career break or to retire.  The factors most likely to motivate retention of pharmacists were reported to be, “Increase in salary and benefits” (84%), “More opportunity to use and develop clinical skills” (73%), “Closer working relationships with other healthcare professionals” (72%) and “More flexible working practices” (70%).  The majority of pharmacists reported perceived levels of under-staffing of both pharmacists and pharmacy technicians. |
| **Assessing Supply in Relation to Prospective Demand for Pharmacists in Ireland**  Report to the Higher Education Authority  Peter Bacon and Associates  1999  Previously available at:  [http://www.hea.ie/uploads/pdf/Pharm%20Report.pdf](http://www.hea.ie/uploads/pdf/Pharm Report.pdf)  (accessed 13.03.2008)  Ireland | A series of interviews with relevant organisations and individuals was conducted and data was collected from the Pharmaceutical Society of Ireland register amongst other appropriate sources.  A brief questionnaire was also used to survey Health Boards and Hospitals to gather an indication as to how many pharmacist posts were filled or vacant in this sector.  B3 | It was suggested that the admission criteria to the pharmacy degree programme was above the level of academic ability required to carry out a career in pharmacy and as a result may lead to pharmacists’ aspirations being unfulfilled.  The number of registered pharmacists did not correlate with the number of pharmacists actually working or available to work in Ireland.  There was an increase in the proportion of younger, female pharmacists registering, however as a large number of these weren’t employed in Ireland therefore the actual age of the workforce was older.  Female pharmacists appeared to have a longer working life than their UK counterparts.  The community sector was the largest employer of pharmacists.  There was a large shortage of hospital pharmacists and many hospitals operated without any pharmacist cover.  An increase in the number of Irish pharmacy graduates by 50 each year was recommended to meet future manpower requirements. |
| **Study of Demand and Supply of Pharmacists, 2000-2010**  Health Care Intelligence Pty Ltd, Australia  February 2003  Available at:  <http://www.guild.org.au/uploadedfiles/Research_and_Development_Grants_Program/Projects/2001-501_fr.pdf>  (accessed 10.03.2008)  Australia | Quantitative and qualitative data obtained from primary and secondary sources. The main source of primary data collection involved two questionnaires mailed to a stratified sample of pharmacists; other sources included interviews with key informants, two workshops with the National Pharmacy Workforce Reference Group and a survey of hospital pharmacy services. Secondary data sources included net migration data, current and future graduate supply, data from the Australian Institute of Health and welfare regarding registration data and distribution of pharmacists between sectors and Australian Bureau of Statistics for labour force data from 1996.  B3 | The supply data estimated a growth rate in the pharmacy workforce of between 1.98% and 2.38% depending on workforce loss by 2010.  An overall demand was predicted to increase from 13,100 full-time equivalent pharmacists in 2000 to 17,200 in 2010.  A shortfall of approximately 3,000 full-time equivalent pharmacists was predicted by 2010.  Five main targets to increase supply were identified; increase the numbers of pharmacists entering the Australian labour workforce from overseas, increase the graduate supply, decrease loss from the workforce, increase the emphasis of labour substitution and further pharmacy rationalisation. |
| **Pharmacy council of New Zealand Workforce Demographics as at 30 June 2007***†  Pharmacy Council of New Zealand  Available at:  <http://www.pharmacycouncil.org.nz/news/documents/WebsiteReportJuly07_000.pdf>  (accessed 25.03.2008)  New Zealand | Information from the public Register of pharmacists as of 30^th^ June 2007 was presented.  C1 | The Register consisted of three parts; practising, non-practising and interns.  The proportion of practising pharmacists had grown by 3% since 2006.  Females comprised 57% of the practising Register.  There was more than double the number of female pharmacists than male pharmacists in the 36-45 age groups. This trend was reversed in the 56-65 age groups.  A very small proportion of the practising Register (0.2%) was recorded as living overseas.  Community pharmacy accounted for 74% of pharmacists’ place of work and hospital accounted for 12%.  There was a 70% reduction in the number of Certificates of Identity issued, which were required to register overseas. This was most likely due to changes in the regulations for pharmacist registration in the UK and Ireland.  The number of pharmacists who registered in New Zealand via reciprocal or legislative agreements numbered 40. |
| **Skilled health professionals’ migration and its impact on health delivery in Zimbabwe**  Abel Chikanda  Centre on Migration, Policy and Society Working Paper No. 4 University of Oxford, 2004  Available at:  [http://www.compas.ox.ac.uk/publications/Working%20papers/WP0404.pdf](http://www.compas.ox.ac.uk/publications/Working papers/WP0404.pdf)  (accessed 18.03.08)  Zimbabwe | A questionnaire survey involving face-to-face interviews was completed by respondents selected from the Ministry of Health and Child Welfare, individual health workers, key informants, migrant and returnee health workers and people who use the health system.  One tertiary hospital, five regional and six district hospitals, two schools of nursing and the Medical School at the University of Zimbabwe were included in the study.  B3 | Data about pharmacist staffing patterns in public health institutions were largely unavailable.  At the beginning of 1997 there were 524 pharmacists registered in Zimbabwe however only 18.7% of the required public health sector posts were filled. This was worsened by recruitment drives from overseas countries, notably the UK.  68% of survey respondents were considering leaving the country; the majority of these, 29%, reported the UK as their preferred destination.  The most common reasons for emigration of healthcare professionals were economic (largely to receive better remuneration) and also to achieve better living conditions.  It was noted by 87% of respondents that public sector salaries were not competitive.  The questionnaire revealed that the main factors motivating the retention of healthcare professionals in Zimbabwe were better salaries, better fringe benefits and a more pleasant and caring work environment.  It was inferred that a proportion of health professionals migrate into the private sector in order to obtain adequate resources to allow migration overseas. |
| **Global Pharmacy Workforce and Migration Report – a Call for Action***  Chan XH, Wuliji T  2006  Available at:  <http://www.fip.org/www2/subsections/index.php?page=menu_resourcesforhealth>  (accessed 09.03.2008)  International | Surveys were sent out to all International Pharmaceutical Federation (FIP) Member Organisations. Three surveys were developed to focus on; pharmacy workforce, Continuing Professional Development (CPD) and Continuing Education (CE) and migration of pharmacists.  B3 | **Pharmacy workforce survey**  34 out of 83 member organisations responded.  There were a greater number of pharmacists per 100,000 in higher income countries. This ratio varied hugely between countries.  Gender imbalances vary geographically; the highest female proportion was in Europe (63%), the highest male proportion was in the Western Pacific/South East Asia region (59%).  The majority of pharmacists worked in the community or hospital with Europe having the highest proportion in community pharmacy, and the Western Pacific/South East Asia having greatest numbers in hospital pharmacy. However, there were large amounts of missing data.  Urban/rural and public/private sector disparities existed in pharmacist distribution. In Central Uganda approximately 90% of the pharmacists serve a quarter of the population.  A significant number of trained pharmacists were unemployed.  In 13 of the respondent countries there was a legal requirement for the certification of all pharmacy technicians.  **Continuing Professional Development and Continuing Education survey**  17 countries out of 34 countries that responded to the workforce survey replied.  Only 3 countries identified having specific CPD systems in place (Japan, UK and Portugal).  CPD/CE is not mandatory in the majority of countries.  Accreditation of CPD/CE providers was mandatory in only three countries.  **Migration of pharmacists**  There was a lack of detailed information regarding migratory data for pharmacists.  Migration affected low income source countries the most.  Greater migration was observed between countries which had reciprocal agreements in place. |
